# Conundrum of Cholesterol Management and Health Implications of Low Cholesterol Levels: A Narrative Review

**DOI:** 10.31729/jnma.9166

**Published:** 2025-07-31

**Authors:** Subodh Bashyal, Manoj Karki, Deepa Shah

**Affiliations:** 1 Universal College of Medical Sciences, Tribhuvan University, Bhairahawa, Nepal; 2 Department of Internal Medicine, Manmohan Memorial Medical College and Teaching Hospital, Kathmandu, Nepal.

**Keywords:** *cholesterol*, *low cholesterol*, *statin*, *optimal cholesterol level*

## Abstract

Lowering cholesterol for the health benefit of dyslipidemia is a well-known fact, but emerging evidence highlights risks associated with excessive lowering. The use of lipid-lowering agents to reduce cardiovascular risk is well-established; however, excessive cholesterol reduction has raised concerns about the potential health implications of excessively low cholesterol levels. This review explores the “cholesterol paradox,” highlighting the dual nature of cholesterol as both a contributor to cardiovascular risk and an essential component for cellular integrity, hormone synthesis, brain function, and vitamin D production. A systematic search of PubMed, Google Scholar, JAMA, and Cochrane Library for eligible studies was conducted from 1980 until December 1, 2024. Evidence suggests that excessively low cholesterol levels may impair cell membrane integrity, neurotransmission, and immune function. This review underscores the need for balanced clinical approach to cholesterol management rather than solely aiming for aggressive reduction, ensuring both cardiovascular protection and broader systemic health.

## INTRODUCTION

Cholesterol plays a crucial role in cardiovascular health and is a key contributor to atherosclerotic diseases.^1^ Hypercholesterolemia is strongly associated with coronary artery disease, stroke, and related conditions.^2,3^ Coronary artery disease alone accounts for over 16% of global deaths.^4^ As a result, cholesterol-lowering therapies have become essential in clinical practice. Over the years, advances in pharmacology have produced highly effective lipid-lowering drugs.^5^ However, maintaining an optimal cholesterol balance is challenging, as excessively low levels may also pose health risks.^6,7^ The overuse of lipid-lowering agents and their impacts on cholesterol levels have become a concern among healthcare professionals.^8^ Often viewed solely as harmful, cholesterol is actually fundamental for cellular structure, hormone production, and overall physiological stability. Its significance extends beyond its role in atherosclerosis. This review examines the complex role of cholesterol, focusing on the impact of extremely low levels and highlighting its broader contributions to human health.

## CHOLESTEROL

Cholesterol, was first introduced by a Poulletier de la Salle in France from bile and gallstones in 1769.^9^ Cholesterol plays a significant role in human physiology by contributing to the structure of cell membranes and serving as a precursor for synthesizing hormones and vitamin D.^10^ Its production is finely regulated through a complex metabolic process involving enzymes, transporters, and receptors.^11^ Beyond its involvement in cellular structure, cholesterol is crucial for generating molecules that regulate various physiological processes.^12^ In cell membranes, cholesterol interacts with phospholipids to maintain stability and flexibility.^13^ Cholesterol is a precursor for vitamin D, various hormones and aids enzymatic conversions in endocrine glands, initiating their synthesis.^14^

## HYPERLIPIDEMIA

Hyperlipidemia is a condition of the human body when there is a high level of circulating lipids in the bloodstream due to genetic or acquired conditions.^15^ This increase in the level of Lipids (Cholesterols and triglycerides) leads to various cardiovascular diseases. The death due to these diseases comprises (17.9 million) 32% of total deaths in the world.^16^ The main source of these lipids is human diet or can be synthesized endogenously.^17^ Cholesterol and triglycerides are transported in blood plasma within large particles, known as lipoproteins, composed of lipids and proteins. There are four major lipoprotein particles: chylomicrons, very low-density lipid protein (VLDL), low-density lipoprotein (LDL), and high-density lipoprotein (HDL). They differ in lipid content, hence density, types of proteins, and different functions.^18,19^ HDL acts to remove excess cholesterol from the circulation to the liver and is therefore beneficial for blood vessel health, unlike the other three.^20^

## LIPID-LOWERING AGENTS

Lipid-lowering drugs are used to treat elevated levels of circulating cholesterol and triglycerides to prevent cardiovascular events, such as ischemic heart disease or stroke. Lipid-lowering therapy aims to reduce the circulating levels of chylomicrons, VLDL, and LDL but increases HDL. Mechanisms of existing drugs include inhibition of cholesterol or lipoprotein production, inhibition of intestinal cholesterol absorption, inhibition of bile absorption to promote cholesterol removal, and promotion of lipoprotein degradation.^21^ Statins are typically known as the first line of therapy as they inhibit the endogenous production of cholesterol.^22^ Statin, widely known for their effectiveness in decreasing cholesterol levels, have emerged as vital tools in reducing the risk of heart disease. The impressive track record of statins in decreasing the likelihood of cardiovascular events has resulted in their widespread use and so rapidly became one of the most commonly prescribed categories of drugs across the globe.^23^ Statins are competitive inhibitors of HMG COA reductions, the enzyme that controls the rate-limiting step in cholesterol synthesis.^24^ As intracellular cholesterol decreases, the cells import more cholesterol from blood plasma to fulfill their needs, effectively lowering plasma LDL levels.^25^ Statins are taken before bedtime because cholesterol synthesis typically occurs during fasting at night.^26^ Common side effects include muscle pain and muscle injury, which may increase with vigorous exercise. Other adverse effects include new development of diabetes and liver damage .^23^

Another class of drugs is bile-binding resins, which are inhibitors of bile reuptake.^27^ Normally, about 95% of bile acids delivered to the duodenum are reabsorbed back into the liver. The 5% that is excreted in feces is compensated by newly synthesized bile in the liver.^28^ Bile-binding resins adhere to negatively charged bile acids in the small intestine, preventing them from being reabsorbed, thus promoting excretion.^29^ As more bile is created in feces, the liver produces more new bile from cholesterol, effectively removing more cholesterol from the blood.^30^ These resins are best taken with meals; common side effects include nausea, bloating, and stomach cramping. In addition, the absorption of fat-soluble vitamins may be reduced, and the production of liver enzymes may also increase.^31,32^ Ezetimibe inhibits the absorption of dietary and Biliary cholesterol from the small intestine without affecting bile when it is taken with meals. Ezetimibe can cause fatigue, diarrhea, headache, runny nose, sore throat, body aches, and some other more serious but less common side effects.^33,34^ Fibrates are agonists for Peroxisome proliferator-activated receptor (PPAR)-alpha. PPAR are transcription factors that mediate the effect of dietary fatty acids on lipoprotein metabolism. Activation of PPAR alpha has several effects: it promotes the degradation of triglyceride-rich particles by lipoprotein lipase, reduces the production of VLDL, and increases the production of HDL by regulating apo apo-lipoprotein A1, the major protein component of HDL.^35,36^ PPAR is usually taken before meals; common side effects include indigestion, abdominal pain, fatigue, dizziness, leg cramps, and increased serum creatinine.^37^ Proprotein convertase subtilisin/kexin (PCSK) inhibitors are monoclonal antibodies that bind to and inhibit PCSK9. PCSK9 is an enzyme that promotes the degradation of LDL receptors, which are required for the cellular uptake of LDL from the blood. Inhibition of PCSK9 increases LDL receptors removing more LDL from blood plasma. These drugs are administered by subcutaneous injections and can cause local injection site reactions, such as erythema pain and bruising, other side effects include hypersensitivity reaction, rhinitis, nausea, and muscular fatigue.^38,39^ Niacin lowers plasma, VLDL, LDL, and triglyceride levels and raises HDL. Significantly proposed mechanisms include decreased triglyceride synthesis and decreased degradation of lipoprotein A1.^40,41^ This drug is taken with meals. Niacin is often poorly tolerated, with many side effects ranging from unpleasant to severe.^42^

## CHOLESTEROL PARADOX

The new understanding of the cholesterol paradox adds a controversial element to the traditional narrative as we explore the complex ground of cholesterol management, especially because of the widespread use of lipid-lowering agents.^43^ While there is no doubt that lipid-lowering drugs like statins are essential in lowering the risk of cardiovascular disease, a significant issue arises: Could the continuous overuse of these drugs for lowering cholesterol levels pose a reciprocating challenge to human health?^44^ The enigma becomes more worrying as new studies reveal the potential major health repercussions like cancers of lowering cholesterol to alarmingly low levels.^45^ In this context, it is crucial to look at both the potential benefits and drawbacks of heart health initiatives. The overuse of lipid-lowering drugs can unknowingly compromise the various facets of health due to our one-way goal of lowering cardiovascular risk.^46^ Exploring the insides of the cholesterol paradox gains particular importance when considering the use of lipid-lowering drug. Overuse or misuse of which may significantly disturb the delicate equilibrium between cholesterol levels and overall health outcomes, as these drugs may unintentionally drive individuals into a state of deficient cholesterol levels.^47^ Low cholesterol levels in the circulation are always linked to disrupted cellular functioning, impacting vital physiological processes that affect cognitive function, the development of cancers, bone metabolism, brain stroke, and overall general health.^48^ This sets up the context for the emergence of the cholesterol paradox. So, lipid-lowering drugs, have to be considered for rational use and adjusted according to the specific needs of the patients.

## LOW CHOLESTEROL LEVELS AND ITS IMPACT ON HEALTH

Lowering cholesterol has always been a hot topic due to its effect on the cardiovascular system and its aid in protection against coronary diseases. Lowering the LDL and VLDL cholesterol is the main aim in the primary and secondary prevention of coronary heart disease.^49^ The protocol for lowering the cholesterol in dyslipidemia patients has a target of lowering the LDL to around 100 mg/dl,^50^ but in the high-risk group, the lipid-lowering agents are more vigorously used to lower the cholesterol level much lower than 70mg/dl.^51,52^ This rapid and tremendous fall in cholesterol levels might have a significant and positive role in cardiovascular health.^53^ However, there is very little concern revealed about the other aspect of lowering cholesterol, which is non-cardiac complications and death due to low levels of cholesterol in circulation.^54,55^ Various research and trials have established that using lipid-lowering agents, even in low LDL or VLDL levels in the body, is cardioprotective and useful for the prevention of coronary heart disease.^56^ However, on the other hand, the fact that low cholesterol levels have various effects on the body is always neglected Figure 2. There needs to be more studies regarding this aspect of the cholesterol level lowering.

## CONSEQUENCES OF THE LOW LEVEL OF CHOLESTEROL IN THE BODY FUNCTION

Cholesterol has a key role in the maintenance of the integrity and permeability of the cell membrane. The cholesterol molecule interacts with the hydrophobic end of the phospholipid, which makes the cell more stabilized and less permeable to small molecules.^13^ So, a decrease in the cholesterol content in the circulation or the human body can harm the cell membrane stability and thus disturb the morphology and proper functioning of the various cells. It is known that low cholesterol disturbs the contour and increases the stiffness of the cells, resulting in disturbing the permeability, fluidity, and strength of the cells.^57^ A previous study found that low cholesterol level was a factor in the stiffening of aortic endothelial cells.^58^ Another study established that low cholesterol was responsible for red blood cell membranes being more likely to change shape, and more oxidation was noted.^59^

Cholesterol is a precursor of the steroid hormone in the human body by complex biosynthesis pathways in the cell’s mitochondria.^60^ Both adrenal and non-adrenal hormones are synthesized utilizing the circulating and de novo cholesterol.^61^ Thus, using lipid-lowering agents such as statins for longer durations and in aggressively high doses may cause disturbances in producing these essential steroid hormones. These lipid-lowering agents may decrease the level of cholesterol in the body as these agents directly affect the synthesis of the cholesterol hormones at the HMG CoA reductase enzyme during the synthesis of cholesterol.^62^

Cholesterol in lipid rafts helps organize ion channels, proteins, and receptors responsible for neurotransmission. This contributes to the effective communication between neurons in the brain.^63,64^ Cholesterol is an important factor for myelin sheet synthesis, without which the synthesis of myelin sheets is impossible. Cognitive function and mood regulation, along with many other neural and behavioral functions of the brain, are linked to estrogen. Some studies have stated a fact that these steroid hormones are mainly sex hormones, and their metabolites are somehow responsible for the release of brain neurotransmitters like Gamma-aminobutyric acid (GABA), dopamine, serotonin, etc.^65^

In recent studies, low cholesterol levels due to the use of lipid-lowering drugs or due to old age are linked to Alzheimer’s and Parkinson’s disease.^66,67^ Low cholesterol is also linked to hemorrhagic stroke in the brain as low cholesterol aids in vascular defects, making it susceptible to easy and rapid breakdown.^68^ Wang et al. in 2013 reported that low cholesterol levels, particularly LDL, were associated with an increased incidence of hemorrhagic stroke.^69^ A clear idea about the relationship between these health conditions and cholesterol has yet to be discovered rigidly. But, the endothelial defect and arterial smooth muscle cell necrosis due to low levels of unoxidized LDL were speculated as the main causes behind this.^70,71^ However, the idea of the usefulness of cholesterol in various brain functions must be addressed when considering these diseases.

A low cholesterol level in the body is associated with Vitamin D deficiency. Cholesterol is the only precursor of vitamin D synthesis, the lack of it will cause the overall deficiency of vitamin D in the body.^12^ The main source of vitamin D is cholecalciferol, also known as pro-vitamin D3, which is synthesized from the 7-dehydrocholesterol, which is a cholesterol precursor present in the epidermis of the skin.^72^ The role of vitamin D in the body ranges from calcium metabolism, bone development, immune function, and inhibition of the parathyroid glands.^73,74^ Besides this cholesterol, specifically, lipoproteins also act as a specific plasma carrier for the vitamins D and E. In this process, LDL and HDL play a crucial role in transporting these vitamins in circulation.^12^ In recent studies, it has been found that low levels of cholesterol in the body, either by the vigorous use of lipid-lowering agents or other causes like old age and malnutrition, lead to a decrease in the total vitamin D level in the body.^75,76^

Cancer itself is a disease with a tie to multifactorial aspects. It is linked to numerous contributing factors, including genetics, environmental exposures, and lifestyle. The relationship of cholesterol with cancer causation can go either way: hyperlipidemia or low cholesterol in the body. There are studies in both directions establishing the fact that high LDL leads to the causation of cancer and low serum cholesterol increases cancer-related mortality.^77,78^ This notion supports the V-shaped relationship of levels of cholesterol with cancer as a disease. However, new concerns have risen as some studies reported that the low levels of cholesterol are related to an increase in total cancer-related deaths.^77^ Another study found that individuals with reduced levels of HDL had a higher chance of cancer development, while others with low total cholesterol level had a significantly lower likelihood of developing cancer.^79^ Hematological cancers like Chronic lymphocytic leukaemia,^80^ and acute lymphoblastic leukaemia,^81^ are also strongly linked with low serum cholesterol levels.

A study have found relationship of low cholesterol level with conditions like digestive dysfunction, infection or sepsis, and impaired endothelial function.^54^ It might sound paradoxical, but low HDL disturbing the vascular endothelium has been found to increase the risk of developing vascular and atherosclerotic diseases.^82^ The endothelial dysfunction is known to result from inflammation in the endothelium due to insufficient cholesterol. Sepsis development and prognosis in severely ill and burn patients are also linked with the low cholesterol in the circulation.^83,84^

## CONCLUSION

Managing cholesterol most involve a cautious and personalized approach due to its multifaceted impact on overall health. The widespread use of lipid lowering drugs has led to the cholesterol paradox. So, the importance of balancing lipid management and recognizing it as an essential element for overall health is undeniable.
